# Protein–RNA interactions important for *Plasmodium* transmission

**DOI:** 10.1371/journal.ppat.1008095

**Published:** 2019-12-26

**Authors:** Kelly T. Rios, Scott E. Lindner

**Affiliations:** Department of Biochemistry and Molecular Biology, The Huck Center for Malaria Research, Pennsylvania State University, University Park, Pennsylvania, United States of America; Children's Hospital of Philadelphia, UNITED STATES

## Translational repression is a common regulatory mechanism used by both transmission stages

Gametocytes and sporozoites must both overcome similar obstacles. After fully maturing, they must lie in wait and be prepared for a fleeting moment when a mosquito takes a blood meal and thus move them between host and vector. After successfully transmitting, they find themselves in a hostile environment that they must exit as quickly as possible or face being digested in the mosquito midgut (gametocytes) or being targeted by antibodies and phagocytes (sporozoites). Because of this, the transmission stages rely heavily on post-transcriptional gene regulation [1], and though this is an energetically costly approach [2], it allows for the required rapid translational responses to environmental changes. Similarly, it is clear that translational repression is also used in a related Apicomplexa species, *Toxoplasma gondii*, for its specialized needs by using orthologous proteins [3, 4]. Together, this underscores the importance of preparedness.

Aside from the transmission stages, it remains controversial whether translational repression is also used in *Plasmodium* asexual stages [5, 6], with earlier studies reporting a discrepancy between mRNA and protein abundance in the asexual blood stages [7, 8], as well as differences in ribosome occupancy compared to peak transcript abundance [9]. However, a more recent report shows that transcription and translation are tightly coupled [10]. Despite this, translational control of *var2csa* has been well documented, showing that expression of an upstream open reading frame (uORF) and the action of the *P*. *falciparum* translation enhancing factor (PTEF) contribute to the translational regulation of *var2csa* [11–14]. More work is certainly warranted to resolve the roles of these control mechanisms in this stage of the parasite’s life cycle.

**Fig 1:**
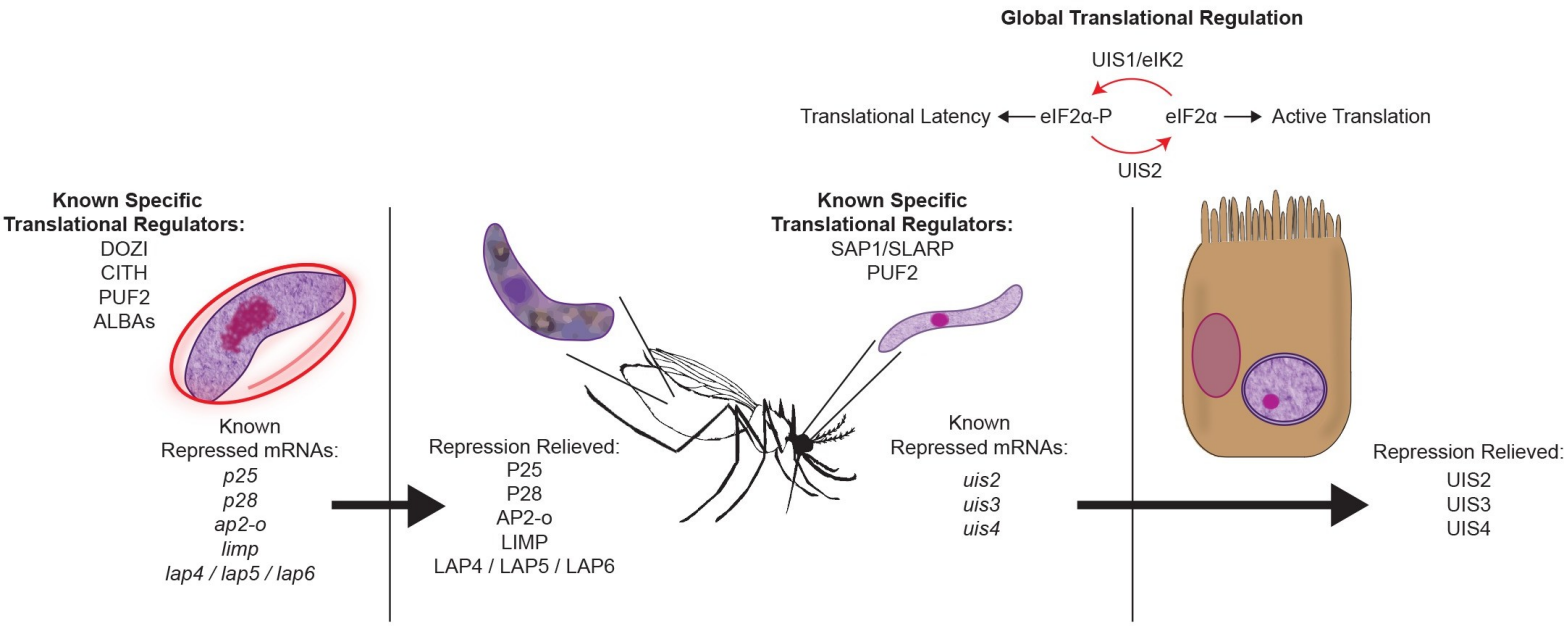
Overview of specific and global translational regulation in *Plasmodium* transmission stages. Specific transcripts are translationally repressed in female gametocytes (left) or salivary gland sporozoites (right) by stage-specific translational regulators. Translational repression of these transcripts is relieved following transmission, and the resulting proteins are essential for proper development and infection of the new host or vector. Global translational repression in sporozoites is controlled by the phosphorylation status of eIF2α by the kinase UIS1/eIK2, which is dominant in sporozoites, and the phosphatase UIS2, which is translationally repressed until the liver stage when it becomes active. eIF2α, eukaryotic Initiation Factor 2α; UIS, Up-regulated in Infectious Sporozoites.

*Plasmodium* parasites, like other eukaryotes, use two tiers of regulation to control the translation of mRNAs in transmission stages: specific translational repression of targeted mRNAs and global translational repression of most mRNAs (regulation in *Plasmodium* depicted in Fig 1, eukaryotic regulation reviewed in [15]). In sporozoites, global translational repression is enacted by a mechanism common to many eukaryotes that involves the regulation of the phosphorylation status of a specific serine residue (S59) on eukaryotic Initiation Factor 2α (eIF2α) by eIF2α kinase (eIK2/Up-regulated in Infectious Sporozoites 1 [UIS1]) to maintain parasite latency [3, 16, 17] and an eIF2α phosphatase (UIS2) to relieve repression following transmission. In contrast, specific translational repression relies upon interactions of RNA-binding proteins with specific mRNAs. In accordance with this possibly elevated role of post-transcriptional regulation, *Plasmodium* parasites have an unusually high proportion of RNA-binding proteins (approximately 10% of the annotated proteome) compared to other eukaryotes [18, 19]. In this scenario, specific transcripts are proactively generated in female gametocytes or sporozoites, which are then bound by RNA-binding proteins and trafficked to cytosolic granules to gain greater stability and to prevent/reduce their translation by the ribosome. While initial evidence for translational repression was seen in targeted studies of individual mRNAs such as *p25* and *p28* [20, 21] and of the DOZI RNA helicase (Development of Zygote Inhibited; an ortholog of DDX6, Dhh1) in gametocytes [22], evidence for the widespread use of translational repression in both transmission stages is now available from comparative transcriptomic and proteomic studies in gametocytes [23] and sporozoites [24–26]. Moreover, our recent study has shown that sporozoites use two orthogonal translational repression programs during their maturation: one that represses mRNAs that encode for host cell traversal and infection functions that is relieved in salivary gland sporozoites and another that represses mRNAs used in early liver stage that is relieved upon transmission [24]. Thus, the use of both global and specific translational repression enables parasite preparedness and is crucial for effective parasite transmission.

## What RNA–protein interactions are important in sporozoites for vector-to-host transmission?

Studies of specific translational repression in sporozoites have focused only on a handful of RNA-binding proteins, in part due to the technical difficulties of working with this parasite stage. Pumilio/FBF 2 (PUF2) and sporozoite asparagine-rich protein (SAP1)/sporozoite and liver stage asparagine-rich protein (SLARP) have been identified as RNA-binding proteins essential for sporozoite transmission and proper development in host hepatocytes, respectively. These proteins each localize to distinct granular complexes in the cytoplasm of salivary gland sporozoites [27, 28], and both contribute to the stabilization of transcripts important for liver stage development, including some of the UIS genes. SAP1/SLARP is essential for sporozoite infectivity and liver infection in *P*. *yoelii* and *P*. *berghei*, as parasites lacking this gene arrest early in liver stage development [29, 30]. Comparative transcriptomic analysis between *pysap1*^*−*^and wild-type salivary gland sporozoites demonstrated that PySAP1 contributes to the preservation of UIS transcripts important for liver stage development [28]. In fact, the deletion of *sap1* in either *P*. *yoelii* or *P*. *berghei* results in greatly reduced UIS transcript abundance, including *uis3* and *uis4*, and *pysap1*^*−*^ parasites do not express UIS3 or UIS4 proteins in liver stage parasites when they are required [29–32]. In contrast, PUF2 is responsible for maintaining the latency and infectivity of salivary gland sporozoites, as *P*. *yoelii* and *P*. *berghei puf2*^*−*^ sporozoites lose infectivity and, remarkably, begin to prematurely dedifferentiate into liver stage-like forms during prolonged residence in the salivary glands [27, 33, 34]. Comparative transcriptomic analysis between *pypuf2*^*−*^ and wild-type salivary gland sporozoites showed that there is large-scale dysregulation in mRNA abundances in the absence of PUF2 that precedes the loss of infectivity and that the parasites can overcome this defect if they transmit in time [27]. PUF-family proteins are known to regulate transcripts by binding to an adenylate-uridylate (AU)-rich PUF-binding element (PBE) that is usually found in the untranslated regions (UTRs) of mRNAs. However, the best studied PUF2-regulated mRNA in sporozoites, *uis4*, appears to be bound and translationally repressed when PUF2 associates with one or more PBEs found in its coding region [35, 36]. Following hepatocyte invasion, the PUF2 granules dissolve [27], and *uis4* mRNA is derepressed [36]. Finally, global and specific translational repression are intertwined in sporozoites, as the mRNA of the UIS2 phosphatase responsible for relieving global translational repression via eIF2α dephosphorylation is regulated by PUF2, thus allowing a stepwise and controlled program of development [17, 37]. While the transcripts for *uis1* and *uis2* are among the most up-regulated and abundant transcripts in salivary gland sporozoites, specific translational repression dictates which activity predominates in sporozoites (UIS1) and liver stages (UIS2). Analogous to the phenotype of *puf2*^*−*^ sporozoites, the deletion of *uis1* results in parasites that prematurely transform into exoerythrocytic-like forms and lose their infectivity [17].

## What RNA–protein interactions are important in gametocytes for host-to-vector transmission?

The infection of a mosquito by *Plasmodium* parasites requires the activation of gametocytes into gametes upon arrival in the midgut of the mosquito and is triggered by extracellular cues, like changes in temperature, the presence of xanthurenic acid, and likely other unappreciated factors [38]. These extracellular changes trigger a signaling cascade propagated by parasite-specific calcium-dependent protein kinases (CDPKs) [39], including CDPK1, which relieves translational repression of transcripts after fertilization occurs in the mosquito midgut [40]. In addition, RNA–protein interactions and post-transcriptional gene regulation are also critical to the infection of mosquitoes and have been easier to study in gametocytes because they are easier to produce/purify en masse and to phenotype in comparison to sporozoites. Two of the major RNA-binding proteins that confer translational repression in female gametocytes are the DEAD-box RNA helicase DOZI (an orthologue of human DDX6 and yeast Dhh1) and the Sm-like factor CITH (homolog of CAR-I and Trailer Hitch; an orthologue of LSM14A) [22, 41]. These two proteins are required for the stabilization of hundreds of transcripts in female gametocytes and are essential for parasite development after fertilization in the mosquito midgut [22, 41]. DOZI and CITH interact with many of the important transcripts that they stabilize, including some of the most highly expressed transcripts in gametocytes, like ookinete surface proteins *p25*, and *p28*, and a mosquito-stage specific transcription factor *ap2-o* [42]. This proper regulation of protein expression of P25, P28, and AP2-O proteins is required for oocyst development in the mosquito [21, 43, 44]. Additionally, DOZI and CITH also translationally repress *limp*, which is translated in the ookinete from maternal mRNA and is a key regulator for adhesion during gliding motility [45]. Finally, coimmunoprecipitation experiments identified additional RNA-binding factors that interact with DOZI and CITH, including translation initiation factor eIF4E, poly(A)-binding protein 1, Bruno/HoBo, Musashi, 7-helix-1, and ALBA-family proteins 1 through 4 [41, 46, 47]. In particular, PyALBA4 plays a role in transcript preservation in gametocytes, including mRNAs from LCCL-domain–containing genes that are important for early mosquito-stage infection, like *lap4*, *lap5*, and *lap6* [47].

Along with their roles in sporozoites, PUF-family proteins also play important roles in female gametocytes. PUF1 was shown to be important for the differentiation and maintenance of *P*. *falciparum* gametocytes [48], whereas PUF2 is important for the regulation of gametocytogenesis in a sex-dependent manner, as *pbpuf2*^*−*^ and *pfpuf2*^*−*^ (but not *pypuf2*^*−*^) parasites make more gametocytes than wild type and have a disrupted male:female gametocyte ratio [27, 33, 49]. And while PUF2 traffics to a complex that is distinct from the DOZI/CITH complex, both complexes have overlapping mRNA targets, like *p25* and *p28* [50]. Moreover, *in vitro* rabbit reticulocyte translation assays demonstrated that PfPUF2 is both the necessary and sufficient *Plasmodium trans*-factor to translationally repress *p25* and *p28* or chimeric mRNAs containing specific PBEs from their UTRs [50].

## Final takeaways and outstanding questions

Translational repression is energetically costly, so there is likely an important reason the parasites use it instead of simply regulating transcription: it allows both readiness and rapid responsiveness to stimuli. However, the use of translational repression allows for an intermediate level of investment: mRNA is made, but the price to produce protein has not yet been paid. Of course, an even more rapid response to stimuli could be achieved by producing the proteins and retaining them in an inactive state. However, this is much more energetically expensive, and the consequences of prematurely expressing (or activating) proteins are dire for transmission stages, as this has been shown to render them noninfectious for both *Plasmodium* gametocytes and sporozoites. Therefore, it is perhaps not surprising that translational repression is subject to multiple tiers of control by both global and specific programs, as well as a diverse and expanded set of RNA-binding proteins that target similar transcripts [19].

While much is now known about how translational repression is used by *Plasmodium* transmission-stage parasites, many outstanding questions remain.

- **What initiates specific translational repression of mRNAs?** This regulation could be imposed from the birth of a transcript in the nucleus by RNA-binding protein association and imprinting, at its receipt by a granule positioned over the exit site of the nuclear pore complex, or even later in the cytosol if a stimulus-specific event switches the program on.
- **How are the mRNA–protein granules organized?** Substantial work has been done to investigate similar cytosolic granules in yeast, which now invokes a nonuniform, liquid–liquid phase separation model that allows for the docking and locking of mRNAs into these granules [51]. Does *Plasmodium* use a similar strategy for one or both of its transmission stages?
- **What are the external stimuli that initiate the dissolution of cytosolic granules post transmission, and what is the mechanism of dissolution?** The most obvious common stimulus is that both gametocytes and sporozoites undergo substantial temperature swings between ambient temperatures and 37°C. However, the directionality of this change is opposite for the two stages, and thus the effect of temperature change upon granule stability would be as well. Could temperature changes instead affect enzymes that can install or remove post-translational modifications (e.g., phosphate groups) that stabilize or destabilize protein–mRNA interactions?
- **Finally, could there be a role for translational repression in male gametocytes as well?** The deletion of genes encoding several RNA-binding proteins also results in male-specific phenotypes, such as an increase, decrease, or ablation of exflagellation. It is feasible that any life cycle stage that requires a rapid response to environmental stimuli could rely upon this strategy.

It is clear that *Plasmodium* has evolved to rely heavily upon post-transcriptional regulation and that many of the same effector proteins are used and repurposed for functionally similar purposes across the life cycle. Future efforts that address the above questions will ultimately help to pinpoint and exploit key weaknesses and could be used to help control parasite transmission.
